# Rapid Development of Microsatellite Markers for the Endangered Fish *Schizothorax biddulphi* (Günther) Using Next Generation Sequencing and Cross-Species Amplification

**DOI:** 10.3390/ijms131114946

**Published:** 2012-11-14

**Authors:** Wei Luo, Zhulan Nie, Fanbin Zhan, Jie Wei, Weimin Wang, Zexia Gao

**Affiliations:** 1 Key Laboratory of Agricultural Animal Genetics, Breeding and Reproduction of Ministry of Education, College of Fisheries, Huazhong Agricultural University, Wuhan 430070, China; E-Mails: lwdjn@live.cn (W.L.); niezhl2004@163.com (Z.N.); zhanfb2012@gmail.com (F.Z.); wangwm@mail.hzau.edu.cn (W.W.); 2 Key Laboratory of Tarim Animal Husbandry Science and Technology, College of Animal Science, Tarim University, Alar 843300, China; E-Mail: weijiedky@126.com

**Keywords:** *Schizothorax biddulphi*, PGM™ sequencing, microsatellite (SSR), polymorphism

## Abstract

Tarim schizothoracin (*Schizothorax biddulphi*) is an endemic fish species native to the Tarim River system of Xinjiang and has been classified as an extremely endangered freshwater fish species in China. Here, we used a next generation sequencing platform (ion torrent PGM™) to obtain a large number of microsatellites for *S. biddulphi*, for the first time. A total of 40577 contigs were assembled, which contained 1379 SSRs. In these SSRs, the number of dinucleotide repeats were the most frequent (77.08%) and AC repeats were the most frequently occurring microsatellite, followed by AG, AAT and AT. Fifty loci were randomly selected for primer development; of these, 38 loci were successfully amplified and 29 loci were polymorphic across panels of 30 individuals. The *H*_o_ ranged from 0.15 to 0.83, and *H*_e_ ranged from 0.15 to 0.85, with 3.5 alleles per locus on average. Cross-species utility indicated that 20 of these markers were successfully amplified in a related, also an endangered fish species, *S. irregularis*. This study suggests that PGM™ sequencing is a rapid and cost-effective tool for developing microsatellite markers for non-model species and the developed microsatellite markers in this study would be useful in *Schizothorax* genetic analysis.

## 1. Introduction

Tarim schizothoracin (*Schizothorax biddulphi* Günther) is an endemic fish species to Xinjiang Autonomous Region, China. It is a cold-water fish species and only distributed in the Tarim River system, which is situated in the arid area in the inland of the Central Asia and possesses unique natural conditions. It had been the main economic fish species in the Tarim River system in the 1960s and once accounted for 80% of total fish catches in the Bostan Lake [1]. However, the population of *S. biddulphi* has declined dramatically since the 1970s because of overfishing, the threat from exotic fishes and numerous water diversions and constructed dams, which prevent the migration of spawning fish [2]. Zhang *et al*. [1] reported that this species was represented by scattered individuals in some rivers and its distribution region became narrow with the number declined greatly as compared to the reported data in 1991. It was rated as Endangered in the 1998 IUCN Red List of Threatened Animals of China in 1998 [3] and considered as the Class II protected species in Xinjiang Autonomous Region in 2004. To protect the genetic resources and develop the breeding stock of this species, studies on its genetic differentiation and population structure are necessary. However, very little genetic resources are currently available for this species.

Microsatellites have emerged as one of the most popular genetic markers for a wide range of applications in population genetics, conservation biology and evolutionary biology. Their codominant nature, high levels of polymorphism, reproducibility and greater information content compared with dominant marker data makes them particularly suitable for the estimation of population structure and genetic diversity [4,5]. However, the major drawback of microsatellite markers in the past has been the high cost of developing species-specific markers [6]. Now, this has been alleviated with the advent of next-generation sequencing, which allows the detection and characterization of SSR loci easily achievable with simple bioinformatics approaches [7]. The random sequencing-based approach to identify microsatellites was rapid, cost-effective and can identify thousands of useful microsatellite loci in a previously unstudied species [6–8]. At present, affordable and fast benchtop high-throughput sequencing instruments like the Ion Torrent Personal Genome Machine™ (PGM™) might enable reference laboratories to switch to genomic typing on a routine basis, which can reduce workload and rapidly provide information for further research.

In the present study, we used the high-throughput sequencing technology PGM™ to obtain a large number of genetic resources for *S. biddulphi* and polymorphic microsatellite loci were subsequently developed. Additionally, the cross utility of these markers was tested in a related, also endangered fish species, *S. irregularis*.

## 2. Results and Discussion

### 2.1. Sequencing by Ion Torrent PGM™

By PGM™ sequencing with a 318 chip, a total of 892.72 Mb data and 3,476,226 quality reads were obtained in a single sequencing run from the genomic DNA of one *S. biddulphi* individual. The length of the reads was quite concentrated in the range of 250 bp to 330 bp, with average of 257 bp. All reads were assembled into 40,577 contigs with mean length of 395 bp (Table 1). A total of 1379 microsatellites were identified in these contigs. SSR’s were found in 3.4% of these contigs and one microsatellite was found every 11.64 kb of genomic DNA (Table 2). Primers were designed for 1016 microsatellites using BatchPrimer 3 (Data S1). Compared to the weeks or even months that can be spent obtaining only tens of microsatellite loci by traditional approaches, the thousands identified here required only one or two days to take the sample from tissue through DNA extraction, library creation and titration and sequencing on PGM™ platform (Table 3). Additionally, the total costs for sequencing was only about $950. The result confirmed that the Ion Torrent PGM™ platform was currently one of the shortest run time and fastest speed [9] and lowest cost next generation sequencers capable of multi-million read level outputs [9,10].

### 2.2. Characteristics of Microsatellites

Among the microsatellites detected, dinucleotides were the most frequent (77.08%), followed by tri- (14.58%) and tetranucleotides (7.47%). Penta- and hexanucleotide SSRs had a much lower frequency (0.65% and 0.22%, respectively) (Table 2). The result was in agreement with most of previous reports on aquatic animals, like *Ictalurus punctatus*[11], *Mogurnda*[12], *Nannoperca*[12], and so on.

**Figure 1:**
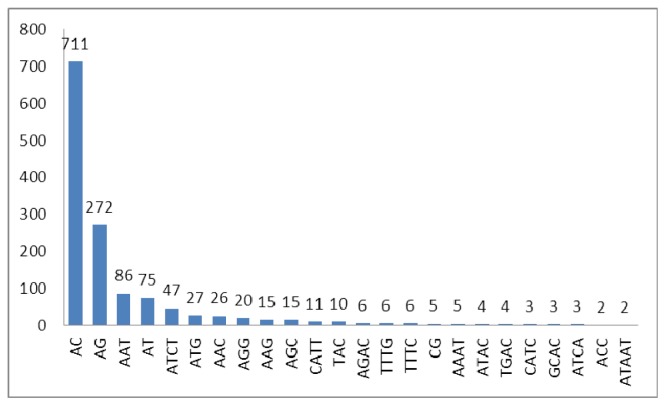
Frequency distribution of microsatellites in *S. biddulphi* based on motif sequence type.

In decreasing order, the 10 most frequently occurring microsatellites were AC, AG, AAT, AT, ATCT, ATG, AAC, AGG, CATT and TAC (Figure 1). The 10 most frequently occurring microsatellites comprised 93.84% of all microsatellites identified. AC is the most frequent motif in *S. biddulphi*, which is the same with *I. punctatus*[11], *Fugu rubripes*[13] and *Etheostoma okaloosae*[14], but different from *Crassostrea virginica* (AG/CT) [15] and *Argopecten irradians* (TA) [16]. The most motifs in aquatic animal are variable, however, GC dinucleotide repeats are extremely rare in all of the genomes studied [17,18], including aquatic animals [11,13,14]. Lower frequencies of CpG dinucleotides in vertebrate genomes have been attributed to methylation of cytosine, which, in turn, increases its chances of mutation to thymine by deamination [19].

### 2.3. SSR Polymorphism

In order to assess the potential use of newly developed microsatellites, 50 random loci were tested for polymorphism in 30 wild individuals of *S. biddulphi*. Of these, 38 loci were successfully PCR amplified and 29 loci were polymorphic across the panel of 30 individuals. The ratio of verified polymorphic markers was 58% in this study, which was higher than those of *Gerris incognitus* (43.5%) [20], *Typha minima* (56.7%) [21] and *Galeorhinus galeus* (40.6%) [22] sequenced by Roche 454. The numbers of alleles detected by the set of 29 polymorphic markers were in the range of 2 to 6 with an average of 3.5 alleles per locus (Table 4). *H*_o_ ranged from 0.15 to 0.83, and *H*_e_ ranged from 0.15 to 0.85. The genetic diversity of *S. biddulphi* in this study was much different from what Gong *et al*. [23] reported. The reasons maybe the different collected SSR loci or samples from different populations. The number of alleles is lower than many other freshwater fish species [15,24] and heterozygosity is mainly concentrating on the middle level. Considering the reduction of its populations, much more attention should be attracted to protect its genetic diversity. There was no evidence for null alleles found in these loci. Four pairs of loci (SCH6 and SCH8, SCH5 and SCH9, SCH5 and SCH10, SCH10 and SCH11) were found to be in linkage disequilibrium and nine of all the 30 loci were deviated from Hardy-Weinberg Equilibrium (*p* < 0.05) (Table 4). A possible explanation for the departure from HWE is the dramatic contemporary decline in spawning populations, and consequent non-random mating and genetic bottlenecks [1,2].

### 2.4. Cross-Amplification in *S. irregularis*

Cross-species amplification was conducted in *S. irregularis.* Out of the 29 SSRs primers tested, 20 (68.97%) were successfully amplified and 13 (44.82%) showed polymorphism in a pilot panel of six individuals in *S. irregularis* (Table 4). The allele number at these 13 loci was ranged from 2–4 with an average of 2.4 alleles per locus. These markers will be useful in *Schizothorax* genetic analysis.

## 3. Experimental Section

### 3.1. Sample and Genetic DNA Extraction

A total of 30 individuals of *S. biddulphi* and six individuals of *S. irregularis* were collected from Tarim River in Xinjiang Autonomous Region, China. Genomic DNA was extracted from alcohol-preserved caudal fin of these specimens by using Phenol/Chloroform procedure [25].

### 3.2. Ion Torrent PGM™ Library Preparation and Sequencing

An Ion Torrent adapter-ligated library was made following the manufacturer’s Ion Fragment Library Kit (Life Technologies, Invitrogen Division, Darmstadt, Germany) protocol (Part #4467320 Rev. A). Briefly, 50 ng genome DNA from one individual was end-repaired, and Ion Torrent adapters P1 and A were ligated using DNA ligase. Following AMPure bead (Beckman Coulter, Brea, CA, USA) purification, adapter-ligated products were nick-translated and PCR-amplified for a total of five cycles. The genome DNA library was purified using AMPure beads (Beckman Coulter) and the quantification, centration and size evaluated by the Agilent 2100 bioanalyzer (Agilent Technologies, Palo Alto, Calif.). Sample emulsion PCR, emulsion breaking, and enrichment were performed using the Ion Xpress Template Kit (Part #4467389 Rev. B), according to the manufacturer’s instructions. Briefly, an input concentration of one DNA template copy and Ion Sphere Particles (ISPs) was added to the emulsion PCR master mix and the emulsion generated using an IKA DT-20 mixer (Life Technologies, Invitrogen division, Darmstadt, Germany). Next, ISPs were recovered and template-positive ISPs enriched for using Dynabeads MyOne Streptavidin C1 beads (Life Technologies, Invitrogen division, Darmstadt, Germany). ISP enrichment was confirmed using the Qubit 2.0 fluorometer (Life Technologies, Invitrogen division, Darmstadt, Germany), and the sample was prepared for sequencing using the Ion Sequencing Kit protocol (Part #4467391 Rev. B). The complete sample was loaded on an Ion 318 chip and sequenced on the PGM™ for 260 cycles. The software CLC Genomics Workbench 5 was used to perform adaptor, poly-A tail trimming and also quality filtering (threshold quality score = 20). Then the reads were assembled to obtain the contigs using CLC Genomics Workbench 5, specifying a minimum read length of 40 nt, a minimum sequence overlap of 40 nt, and a minimum percentage overlap identity of 80%. The trimmed reads were submitted to NCBI Sequence Read Archive under the accession number of SRA059449.

### 3.3. Mining SSR Loci and Primer Design

The simple sequence repeat regions (SSR) were mined among the contigs using the BatchPrimer3 software [26], and the criterion was set for detection of di-, tri-, tetra-, penta- and hexa-nucleotide motifs with a minimum of 6, 5, 5, 5 and 5 repeats, respectively. Primers flanking of the microsatellites were designed using BatchPrimer 3 software and primer sequences for microsatellites are listed in Supplementary Table 1. According to Jurka’s [27] method with minor changes, SSR composed of tandemly repeated basic units 2–6 nt/bp long. As a result of theoretically possible, dinucleotide contains four kinds (AT, AG, AC and GC), trinucleotide contains 10 kinds (AAT, AAC, AAG, ATC, ACG, ACT, AGC, GCC, AGG and ACC), tetranucleotide contains 33 kinds, pentanucleotide contains 102 kinds and hexanucleotide contains 350 kinds.

### 3.4. PCR Amplification and Genotyping

Randomly, 50 microsatellites were selected to test the polymorphism. All SSR primer pairs were synthesized by Invitrogen Co. (Shanghai, China). The reagents for PCR amplification were bought from Tiangen Biotechnology Co. Ltd. (Beijing, China). All the amplifications were carried out in a 10 μL volume containing 1 μL 10× buffer (with Mg_2+_) for Taq DNA polymerase, 100 μM dNTP, 0.5 μL primer pairs, 1 U Taq DNA polymerase and 50 ng genomic DNA. The program of reaction was 5 min at 95 °C, followed by 30 cycles of 30 s at 94 °C, 30 s at optimized annealing temperature (Table 4), 30 s at 72 °C and a final extension at 72 °C for 8 min; at last storing at 4 °C. The PCR products were separated by 1% agarose gel electrophoresis with voltage of 90 V lasting about 20 min. PCR products were separated by electrophoresis on 8% non-denaturing polyacrylamide gels with voltage of 150 V lasting 2 h and visualized via silver-staining.

### 3.5. Data Analysis

The number of alleles (*N*_a_), the effective number of allels (*N*_e_), expected (*H*_e_) and observed heterozygosities (*H*_o_) were calculated using POPGENE 32 software [28]. Deviations from Hardy–Weinberg equilibrium (HWE) for each locus, linkage disequilibrium (LD) between all loci were tested by online version GENEPOP (http://genepop.curtin.edu.au/) [29]. All results were adjusted for multiple simultaneous comparisons using a sequential Bonferroni correction. The presence of null alleles was checked by MICRO-CHECKER version 2.2.3 software [30].

### 3.6. Microsatellite Markers Cross-Amplification in *S. irregularis*

To determine the potential for cross utility, amplification of the identified markers was assessed in one related species, *S. irregularis*, also an endangered freshwater fish species without effective molecular maker.

## 4. Conclusions

Taken together, our first experience with the use of Ion Torrent PGM™ for genome sequencing of fish was very positive with respect to speed, accuracy and cost. It proved that it is an efficient way to develop SSR markers with the application of PGM™, even though some items like read length and accuracy of assembly need to be improved. Additionally, much more attention should be attracted for the protection of the genetic diversity of this endangered fish species. The newly developed microsatellite markers would be useful for its further conservation genetic studies.

## Figures and Tables

**Table 1:** Summary of PGM™ sequencing and assembly.

Sequencing	Total number of bases (Mbp)	892.72
	Total number of reads	3,476,226
	Mean length of all reads (bp)	257
	Longest read (bp)	399

Assembly	Total number of contigs after assembly	40,577
	Mean length of contigs (bp)	395

**Table 2:** Frequency distribution of different repeat type (2–6 motif units) microsatellites identified in contigs from PGM™ sequencing of genomic DNA from *S. biddulphi.*

Repeat	Number of loci identified	Percentage (%)	Frequency (%)	Mean distance (kb)
Dinucleotide	1063	77.08	2.62	15.11
Trinucleotide	201	14.58	0.50	79.89
Tetranucleotide	103	7.47	0.25	155.90
Pentanucleotide	9	0.65	0.02	1784.16
Hexanucleotide	3	0.22	0.01	5352.48
Total SSRs	1379	100	3.40	11.64

Note: Frequency = SSR number/total number of non-redundant sequences; Mean distance = Total length of non-redundant sequences/total SSR number.

**Table 3:** The time, costs and infrastructure needed for the library prep, sequencing of PGM™ and bioinformatics pipeline.

Step	Run time (h)	Cost (dollar)	Instructures or softwares
Ion Torrent adapter-ligated library preparation	7–11	$100	Common molecular biology equipment
Sample emulsion PCR and enrichment	4–6.5	$150	One Touch V26
Sequencing with a 318 chip	4.5–5.5	$600	Ion Torrent V2.0
Sequence assembly	5–8	$100	CLC Genomics Workbench 5
SSR Mining and primer design	5–8	free	BatchPrimer 3 software
Total	25–39	$950	

**Table 4:** Characteristics of 30 polymorphic microsatellite loci isolated from *S. biddulphi.*

Locus/GenBank Accession No.	Primer sequence(5′→3′)	T_a_ (°C)	Repeat motif	Size range (bp)	*N*_a_	*H*_o_	*H*_e_	*P*_HWE_	*N*_a_ in *S. irregularis*
SCH1/JX473024	F: GCCATCCTTCAGTTGTGTCT	62	(TATC)_7_	240–288	6	0.70	0.83	0.00 *	4
	R: AACCGAGTTTCATCCTCCTT								
SCH2/JX473025	F: CTATGCTCGGTTTCTTTTCA	57	(CA)_13_	130–144	3	0.40	0.59	0.04 *	2
	R: ACTGATGTGTGTGTGCGTGT								
SCH3/JX473026	F: ATCCACGCTCTCACACTCTT	59	(GT)_26_	192–214	3	0.75	0.54	0.15	1
	R: CCAGCTCCTCAACACAGATG								
SCH4/JX473027	F: GTGTGTGTGTGCGAGAGTGT	54	(TG)_10_	211–231	3	0.60	0.59	0.11	2
	R: TTCAGATGTAACCCCCTTTG								
SCH5/JX473028	F: TGAAAGTTCCTTTGCTCCTG	52	(TG)_10_	188–234	3	0.70	0.62	0.00 *	2
	R: GTGACACACTGTGCAAAAGC								
SCH6/JX473029	F: GTGTGTGTGTGTGTGTGTGTG	59	(TC)_12_	130–156	4	0.75	0.66	0.17	-
	R: CCATTACGCCTATGGAATGT								
SCH7/JX473030	F: GTGGGGTGATGGAAAATACA	59	(GT)_11_	190–222	3	0.75	0.62	0.56	1
	R: GGCTGACCATTGTGCTAAAC								
SCH8/JX473031	F: AAGGTTGAACAGTTGTTTGC	54	(TTA)_7_	105–125	3	0.75	0.61	0.04 *	-
	R: ATGTCCAGTGTAGCGACTGA								
SCH9/JX473032	F: GTGCAGCTCTGTCTCGATCT	57	(TG)_13_	218–240	3	0.35	0.52	0.17	-
	R: TGTGGATTGTTGCAGTGTTT								
SCH10/JX473033	F: TTCATTGTTGCATTCCTTCC	51	(TTCC)_5_	192–234	3	0.75	0.61	0.07	3
	R: GTTGGTGATGGTGTTCTGCT								
SCH11/JX473034	F: CGGCAACCAGACCGTGTA	50	(GT)_15_	190–214	4	0.50	0.52	0.51	2
	R: CTCCCATACCGCTCCTCC								
SCH12/JX473035	F: TAAAATCGAAGGGGAACA	50	(TG)_15_	151–183	3	0.70	0.66	0.12	2
	R:GACAGTGAGAAGAGGAAACA								
SCH13/JX473036	F: TTTCCCCTTAGTCATTTC	50	(AG)_10_	100–124	3	0.55	0.62	0.03 *	-
	R: GGTGTTTGTCAGGAGTTG								
SCH14/JX473037	F: TTATCTGGACGGAGTGAA	50	(AAC)_8_	189–215	4	0.70	0.70	0.88	2
	R: CATTTTGGGGTGAACTAT								
SCH15/JX473038	F: TCGGTCAATGATGGTGTT	54	(ATA)_8_	238–268	4	0.80	0.67	0.58	-
	R: TTTGGCAGGTCCTTCTTA								
SCH16/JX473039	F: CACAGATAAGAACACGAAT	50	(CA)_23_	242–288	3	0.15	0.27	0.02 *	1
	R: AGGGTTTGGAAGAGGTA								
SCH17/JX473040	F: ACTATTTGTGAGCAGCCC	59	(GA)_15_	343–369	3	0.58	0.57	0.42	-
	R: TATGCGGAAAACCGTGAC								
SCH18/JX473041	F: TCAATGAGCAACGAAAGAGC	52	(AGGCAG)_5_	136–176	4	0.75	0.63	0.00 *	1
	R: ATGGTGGCGAAGGGAGAA								
SCH19/JX473042	F: ACACTCCTGCTACGGTCA	57	(TGA)_5_	446–482	3	0.42	0.68	0.10	1
	R: TACATCGCCTCTGCTCCT								
SCH20/JX473043	F: CGCCAGCGTCTGCCACAA	58	(AGC)_5_	220–316	6	0.64	0.75	0.11	4
	R: GCCGCCATCTTCACCCAC								
SCH21/JX473044	F: TGCCTCAAGGAACTGGTG	50	(CGACG)_5_	154–174	2	0.39	0.39	0.97	2
	R: GAGCATTAGAGTATCGTGGT								
SCH22/JX473045	F: CCGTGGTAAGCACAAGAG	54	(CAAA)_5_	340–362	3	0.63	0.48	0.30	2
	R: GACAGCAGGAGGAGAAGG								
SCH23/JX473046	F: TGACGGTAGAGTCCAGTG	50	(CAATTC)_5_	162–184	2	0.74	0.50	0.03 *	-
	R: TGTAATGACGAACAAGCA								
SCH24/JX473047	F: GACACTGCGTTTTGAAGG	50	(AG)_11_	146–168	3	0.72	0.60	0.33	-
	R: GTCTAACCAGTCGCTCCA								
SCH25/JX473048	F: CCCAGTTACAGCCTTCTC	46	(AC)_12_	195–217	2	0.30	0.51	0.06	1
	R: CAGTTAGTTAGTAGGATGCG								
SCH26/JX473049	F: ACACTAATAAGCATCAGCAG	56	(AC)_10_	147–175	5	0.50	0.64	0.26	1
	R: CACAGTTCACAAGAGCAAG								
SCH28/JX473051	F: ATGAGAGCAGAAGAGTGGG	50	(TG)_10_	262–334	4	0.85	0.71	0.58	2
	R: GAGGAGGCTGTGAGGAAC								
SCH29/JX473052	F: TTAGAAGTGGAGACAGTT	56	(TG)_11_	260–334	6	0.85	0.78	0.05	3
	R: TGAGAGTAAAGAGAGAGC								
SCH30/JX473053	F: TACTTTCTATCGTGTTTTTG	48	(TA)_8_	148–164	4	0.45	0.46	0.83	-
	R: GTAACCTGCTGAACTTTG								
Mean value				3.5	0.611	0.598			2.4

*N*_a_: observed number of alleles per locus; *H*_o_: observed heterozygosity; *H*_e_: expected heterozygosity; *P*_HWE_: probability value by Markov chain method for the Hardy–Weinberg equilibrium;

* denoted significant departure from HWE after Bonferroni correction (*p* < 0.05).
